# Influence of Intended Slow and Fast Eccentric Back Squat Velocity on Subsequent Countermovement Jump Performance

**DOI:** 10.3390/jfmk11020143

**Published:** 2026-03-31

**Authors:** Artemis Zarkadoula, Themistoklis Tsatalas, Anthony D. Kay, Anthony J. Blazevich, Christos Kokkotis, Spyridon Plakias, Brett Anthony Baxter, Alex J. Van Enis, Giannis Giakas, Minas A. Mina

**Affiliations:** 1 Department of Physical Education and Sports Science, University of Thessaly, 42100 Trikala, Greece; azarkadoula@uth.gr (A.Z.); ttsatalas@uth.gr (T.T.); spyros_plakias@yahoo.gr (S.P.); 2 Sport, Exercise and Life Sciences, University of Northampton, Northampton NN1 5PH, UK; tony.kay@northampton.ac.uk (A.D.K.); brett.baxter@northampton.ac.uk (B.A.B.); 3 Centre for Precision Health, School of Medical & Health Sciences, Edith Cowan University, Joondalup, WA 6027, Australia; a.blazevich@ecu.edu.au; 4 Department of Physical Education and Sports Science, Democritus University of Thrace, 69100 Komotini, Greece; ckokkoti@affil.duth.gr; 5 Sport and Exercise Science, School of Health, Sport and Rehabilitation, University of Derby, Derby DE22 1GB, UK; a.vanenis@derby.ac.uk

**Keywords:** warm-up exercise, conditioning exercise, resistance training, vertical jump

## Abstract

**Background**: The back squat is a key strength and conditioning exercise used to develop lower-limb strength and power, yet little is known about how movement velocity influences its acute performance-enhancing effects, such as improvements in countermovement jump height and power. The present study examined the acute effects of slow (v_slow_) versus fast (v_fast_) eccentric-phase velocity during back squats performed withmaximal concentric velocity on subsequent countermovement jump (CMJ) performance, using a randomized, crossover design. **Methods**: Fourteen male subjects (age = 22.9 ± 1.9 years; height = 1.8 ± 0.1 m; mass = 76.4 ± 8.3 kg) visited the laboratory on two separate days and completed a comprehensive task-specific warm-up followed by three v_slow_ or v_fast_ back squats at 70% of one-repetition maximum. Three CMJs were performed before and 30 s, 4 min, 8 min, and 12 min after the interventions. Jump height, peak power, kinetic energy, maximum knee angle, and knee angular velocities in both eccentric (downward) and concentric (upward) phases were recorded. **Results**: No significant (*p* > 0.05) between-condition differences were detected in any measure. Compared to pre-intervention, significant increases (collapsed data) were detected in jump height (6.0%; *d =* 0.68–0.83), power (3.6–6%; *r =* 0.32–0.38), and kinetic energy (5.0–8.0%; *d =* 0.62–0.86) at 30 s and 4 min. **Conclusions**: Given the lack of between-condition differences, the eccentric movement velocity of moderate conditioning back squats with maximal concentric velocity exercises does not appear to influence subsequent jump performance enhancements. Thus, either conditioning activity can be used to improve subsequent jump performance. However, as performance was enhanced only at 30 s and 4 min post-intervention, the window of opportunity is narrow, and timing should be carefully considered when including such activities in pre-competition routines.

## 1. Introduction

Warm-up routines are typically designed to condition the neuromuscular system to acutely enhance voluntary force production during subsequent high-intensity physical activities [1,2]. The resulting mechanical power output improvement is often mischaracterized as a classic post-activation potentiation (PAP) response. However, PAP refers to a specific intracellular mechanism, typically observed as an increase in electrically evoked muscle twitch force production [3,4] rather than maximal voluntary force production [3]. To address this distinction, the term post-activation performance enhancement (PAPE) has been used to describe short-term improvements in voluntary muscular performance (e.g., countermovement jump [CMJ] height) following traditional warm-up routines that involve cardiovascular, stretching, and skill-specific exercises [1]. Similarly, the inclusion of high-intensity movement tasks, often termed conditioning activities (CA), has also been reported to induce jumping and sprinting performance [3,5,6,7]. This distinction in terminology is of particular practical importance as the design of warm-up strategies can influence PAPE [5,8,9,10] prior to training or competition, with the optimal components of warm-up protocols still unclear.

A growing body of research has confirmed additional performance enhancements (e.g., CMJ height, squat 1-RM) beyond those of a traditional warm-up following high-intensity CA [5,11,12]. A recent systematic review [1] indicated that load was important, as using barbell exercises with heavier conditioning loads (85–90%) of one-repetition maximum (1-RM) resulted in greater performance enhancements in subsequent high-velocity voluntary tasks (e.g., squat jump, CMJ, and drop jump) than lighter loads (65–70% 1-RM). This finding indicates that load may be an important component. However, as movement and thus muscle contraction velocity should be slower under heavier load conditions, it remains unclear whether the observed effects result from the increased load or the resulting reduced velocity. CAs are recommended to be performed with maximal intent in the concentric phase to maximize performance gains [13,14,15]. However, movement velocity affects time under tension as well as the load lifted [16], with movements performed at a slower velocity prolonging time under tension, potentially increasing fatigue, and acutely decreasing power output [17]. Thus, as a greater load may reduce maximal concentric velocity, intentionally slowing the eccentric phase of the squat would likely also result in decreased maximal concentric velocity, potentially delaying or mitigating PAPE effects. A 4-s eccentric duration can alter the mechanical stimulus by increasing time under tension compared to faster contraction speeds, as shown by Wilk et al. [18] who demonstrated that a 4-s duration provided a different physiological environment compared to faster, traditional movements by increasing cardiovascular demand (e.g., greater heart rate and blood pressure responses), elevating metabolic stress (reflected by higher lactate accumulation), and amplifying acute hormonal responses, particularly growth hormone concentrations. Slower durations (2–4 s) can elicit significant PAPE effects according to Wilk et al. [19], provided the recovery interval is sufficient to offset the increased time under tension. However, to date, limited research has compared different movement velocities during squatting on subsequent dynamic performances (e.g., CMJ). Although CAs are recommended to be performed explosively in the concentric phase to maximize performance gains [13,14,20], there is still debate over the optimal exercise load and velocity of eccentric contractions required to maximize the potentiation magnitude.

There are a number of mechanisms that can explain PAPE, such as changes in muscle temperature, with subsequent performance improvements of 5–10% possibly induced by temperature increases alone [3,21]. Increases in muscle temperature of ~1–2 °C are associated with meaningful improvements in power output and can occur within several minutes of active muscle loading, where contraction durations are typically in the range of ~2–4 s per repetition [22,23]. A recent systematic review and meta-analysis by Wilson et al. [24] reported that high-speed (velocity-dependent) tasks improve by ~3.7% per °C increase in muscle temperature, with increases of 2–3 °C commonly observed following vigorous exercise; however, more modest temperature elevations are also frequently attained after shorter bouts of activity. This suggests that even relatively small increases in muscle temperature (e.g., ~1–2 °C) following a conditioning activity may meaningfully enhance subsequent CMJ performance. Slow eccentric contractions that ensure active muscle engagement throughout the lengthening phase, thus facilitating the controlled dissipation of kinetic energy into heat [25,26,27], may therefore be beneficial. The resultant localized increase in muscle temperature may improve muscle function, possibly enhancing concentric performance following eccentric conditioning protocols [28]. Furthermore, slow eccentric contractions may offer unique benefits in training due to their extended duration, which allows more time for sensory feedback and motor learning to occur [29]. Douglas et al. [30] highlights that slower eccentric contractions, typically lasting ~2–4 s compared with <1 s in faster conditions, result in greater afferent feedback from mechanosensitive receptors due to prolonged muscle-tendon strain and tension development. This prolonged eccentric phase enables the central nervous system to refine movement patterns and improve technique through eSnhanced proprioceptive input [30]. Consequently, the thermal and neuromuscular benefits associated with slow eccentric contractions may play a distinct role in preparatory or rehabilitative phases of training, where enhancing control, feedback, and tissue readiness are key objectives.

Nonetheless, whilst reducing movement velocity during the eccentric phase may induce beneficial thermal and proprioceptive responses to enhance PAPE, it will likely induce greater fatigue and thus compromise the resultant maximal concentric velocity available to an individual. Nuzzo et al. [31] reported that rapid force production during the eccentric phase of a movement requires high and fast muscle activation, especially in athletic tasks involving fast, light stretch-shortening cycles (SSC) such as sprinting and plyometrics. Such movements necessitate rapid deceleration and reversal of momentum, increasing the maximum force during the exercise, which is typically produced at the eccentric-concentric transition point where agonist muscle lengths are longest and induces a momentary “eccentric overload” that is not available during movements with slower eccentric velocities [32]. This high-velocity force production can improve neural drive and facilitate more effective motor unit recruitment and coordination, aligning with the principle of training specificity, which is achieved when the stimulus replicates the velocity and force characteristics of the target performance activity [31]. Additionally, faster contractions may minimize fatigue that would otherwise result from the greater time under tension associated with slower contractions. Nonetheless, the later but more rapid muscle activation during the eccentric phase of fast SSC activities causes a muscle-tendon behavior in which the tendon stretches under increasing load while, the muscle remains quasi-isometric, or even shortens, thereby minimizing actual eccentric muscle lengthening [33]. This would limit the conversion of chemical energy to heat and may thus compromise muscle temperature-derived enhancements in performance. Thus, the influence of contraction velocity on potential PAPE contributors is potentially contradictory, and consequently, the influence of contraction velocity on performance outcomes remains unclear.

Given the arguments above, it is important for strength and conditioning coaches to determine whether manipulating movement velocity in the eccentric squat CA may influence jumping performance. Therefore, the purpose of the present study was to examine and compare the effects of intended slow versus fast eccentric velocities during back squat exercises (with maximal velocity during the concentric phase) on subsequent CMJ performance at different post-conditioning time points (i.e., 30 s, 4 min, 8 min, and 12 min). It was hypothesized that (1) fast (v_fast_) and slow (v_slow_) eccentric velocity squat CAs would increase subsequent CMJ height and alter CMJ parameters (i.e., peak power, peak eccentric kinetic energy, peak knee flexion angle, and angular velocities), and (2) the slow (v_slow_) eccentric velocity squat CA may result in greater improvements in subsequent CMJ height than the fast (v_fast_) CA.

## 2. Materials and Methods

### 2.1. Study Design

A randomized, crossover study was designed to compare CMJ performance following two PAPE protocols: intended slow-velocity (v_slow_) or intended fast-velocity (v_fast_) movement during the descending phase (hereafter, the “eccentric” phase) of the back squat exercise. Both protocols were performed following a comprehensive, task-specific warm-up to determine how these may influence the magnitude and time course of changes in subsequent CMJ performance. To test these hypotheses, 3D motion analysis was used to record knee angle and peak knee angular velocities, while synchronized force platforms were used to record ground reaction forces to calculate maximal countermovement jump height, peak power, and kinetic energy. The subjects visited the laboratory on three occasions at the same time of day, each separated by 72 h. The subjects were familiarized with the experimental testing protocols, and their 1-RM back squat load was determined. They then visited the laboratory on two further occasions, under two experimental conditions (v_slow_ and v_fast_ interventions), in a randomized, counterbalanced order. In the experimental conditions, a comprehensive task-specific warm-up (described below) was performed, followed by three CMJs to assess pre-intervention performance, followed by the v_slow_ or v_fast_ conditioning activity (three back squats at 70% 1-RM). CMJ trials were then performed 30 s, 4 min, 8 min, and 12 min after the interventions to determine the influence of the interventions on jump performance.

### 2.2. Subjects

For this repeated-measures, within-subject study, a convenience sample of 14 active males (age = 22.9 ± 1.9 years, height = 1.8 ± 0.1 m, mass = 76.4 ± 8.3 kg) with ≥5 years of experience in weight training and no recent illness or lower-limb injury volunteered to participate, and then completed all measurements, after completing a written informed consent and a pre-test medical questionnaire. Both males and females were invited to participate; however, only males volunteered. The subjects were instructed to maintain normal dietary habits throughout the study and to avoid strenuous exercise and stimulant use for at least 72 h prior to testing. Ethical approval was granted by the ethics committee at the University of Thessaly, Greece (approval number 2-1/7 June 2023), with the study conducted in accordance with the Declaration of Helsinki.

Based on data from similar studies [34,35], moderate effect sizes (ES [Cohen’s *d*]) were calculated from mean changes in jump height (ES = 0.70) and mechanical power output (ES = 0.67). As the analysis employed ANOVA, Cohen’s *d* (*d* = 0.70) was converted to Cohen’s *f* (*f* = 0.35) for use in power analysis calculations. A priori power analysis was conducted using G*Power version 3.1.9.7 [36] to determine the minimum required sample size to detect statistically significant differences. The analysis was conducted for within-between interaction in a two-way repeated measures ANOVA using the following parameters: statistical power 1 − β error (power) = 0.80 (80% probability of detecting a true effect, corresponding to a 20% Type II error), using α = 0.05 (5% Type I error), and ES = 0.35 (Cohen’s *f*). The power analysis revealed that a minimum sample of 12 subjects was required, with 18 recruited to account for possible data loss and subject attrition. Four subjects did not complete all testing protocols; hence, statistical analyses were conducted on complete data sets from 14 subjects, which exceeded the minimum required sample size for statistical power.

### 2.3. Procedures

#### 2.3.1. Overview

The subjects were familiarized with the experimental testing protocols and had anthropometric characteristics and 1-RM back squat load recorded 1 week prior to data collection. They then visited the laboratory on two further occasions under two experimental conditions (v_slow_ and v_fast_ interventions), with each experimental session separated by 72 h and performed at the same time of day. During experimental conditions, following the comprehensive task-specific warm-up (described below), the subjects performed three CMJs to assess pre-intervention performance, followed by the v_slow_ or v_fast_ conditioning activity (three back squats at 70% 1-RM). CMJ trials were then performed 30 s, 4 min, 8 min, and 12 min later to determine the influence of the interventions on jump performance. The selected time points were intended to sample early (30 s), short (4 min), moderate (8 min), and extended (12 min) recovery windows, with sufficient between-test intervals to minimize interference effects, account for inter-individual variability in the balance between potentiation and fatigue, and increase the likelihood of identifying the time frame at which performance enhancement may occur [9].

#### 2.3.2. Familiarization Session and One-Repetition Back Squat Lift Test

Back squat 1-RM load was assessed using standard protocols [37]. Subjects initially performed a 7-min jogging warm-up at 7.5 km·h^−1^ on a treadmill (Technogym S.p.A., Cesena, Italy). Two minutes later, five unloaded squat repetitions were performed at a 2 s/2 s (eccentric/concentric) rhythm, which were repeated after 30 s rest using a 1 s/1 s rhythm. The subjects then completed 8–10 squat repetitions at 50% estimated 1-RM before the load was increased by 20% for 3–5 repetitions, and by a further 20% for 2–3 repetitions, with 2 min rest between sets. The load was finally increased by 5% with 2–4 min rest between single lifts until subjects failed to complete the lift; the previous successful attempt was recorded as their 1-RM. To ensure correct technique, the bar was positioned above the posterior deltoids at the base of the neck, with the feet shoulder width apart, the toes pointed slightly outward, and the subjects squatting to ~90° knee angle before returning to a standing position. To ensure a similar squat depth, a box was placed behind the subjects to limit knee flexion to ~90°. All subjects received strong verbal encouragement to promote maximal effort, and spotters were used to ensure subject safety.

#### 2.3.3. Comprehensive Task-Specific Warm-Up and Countermovement Jump Trials

In the experimental trials, subjects performed a comprehensive task-specific warm-up, identical to the protocol used by Mina et al. [5]. The warm-up was task-specific to the CMJ testing task, incorporating progressive CMJ efforts alongside squat-pattern movements to closely match the biomechanical and neuromuscular demands of the subsequent performance assessment. This consisted of a 5-min jog at 7.5 km·h^−1^, followed by five continuous unloaded squats (non-jumping) at a 2 s/2 s rhythm, then unloaded squats at a 1 s/1 s rhythm after 30 s of rest, and then five continuous CMJs at ~70% of the subject’s perceived maximum after a 20-s rest. After a further 30 s rest, maximal CMJs were performed every 30 s until three consecutive jumps were performed within 3% of jump height (4–7 jumps were performed in all trials), and thus additional practice was unlikely to evoke further improvement. CMJs were initiated from a stationary upright standing position with hands on the chest, making a preliminary downward movement to a self-selected depth with the hips and knees flexed, and immediately jumping vertically as high as possible [5].

Two minutes following the completion of the task-specific warm-up, subjects performed three maximal pre-intervention CMJ trials to establish baseline (i.e., after warm-up) performance. A conditioning set of three repetitions of back squats at 70% of their previously determined 1-RM load was performed using either v_fast_ or v_slow_, followed by three CMJs at 30 s, 4 min, 8 min, and 12 min after conditioning (see Table 1). The post-intervention intervals were selected from previous studies describing the time course of the performance augmentation (PAPE) response [5,14].

#### 2.3.4. Conditioning Activities

In both conditions (v_fast_ and v_slow_), the load was set to 70% 1-RM, with the subjects performing one set consisting of three back squat repetitions. In the v_fast_ condition, all subjects performed the eccentric and concentric phases at their maximum volitional velocity. In v_slow_, subjects squatted down at a slow velocity as indicated by a metronome-guided movement tempo (Korg MA-30, Korg, Melville, NY, USA) [19] taking 4 s to complete the eccentric phase, holding the lowest position for approximately 1 s, and then performed the concentric phase with maximum effort, and thus, maximum velocity.

#### 2.3.5. Kinetic and Kinematic Analyses

Kinematic data were collected during the CMJ using a Vicon motion analysis system (T-Series, Oxford Metrics LTDA, Oxford, UK) with 10 cameras operating at 100 Hz surrounding two force platforms (Bertec, FP4060-10-2000, Bertec Corporation, Columbus, OH, USA). Ground reaction forces were sampled at 1000 Hz and synchronized with the Vicon system. The data were then filtered using Woltring’s quantic spline algorithm with a mean squared error setting of 15 [38] before running the Plug-In-Gait biomechanical model (Vicon Plug-in-Gait, Oxford Metrics). The procedures identified by Davis et al. [39] were followed to define Cardan angles and to reconstruct a system of embedded coordinates from the marker set to 0° at the hip, knee, and ankle in a standing position. Lower-limb kinematic data were captured by placing 16 reflective markers over the pelvis, left and right thigh, left and right shank in a straight line, and the left and right foot at a right angle to the leg [40]. Data were analyzed using Vicon Nexus (v2.18) software to determine peak hip, knee, and ankle flexion angles and angular velocities during the CMJ trials.

All jumps were performed from the standing position, with each foot in parallel, on two force platforms, providing separate time-synchronized measurements of force data for each leg. The subject’s body weight was calculated by summing the signals from the force platforms for each leg and averaging the summed vertical force when the subject was stationary using an average window of 500-ms. Subsequently, the force of body weight was subtracted for each time point of the entire signal. Following methods by Mina et al. [5] jump initiation (i.e., the beginning of the downward, or eccentric, phase) was identified as the point at which the ground reaction force (N) decreased by two standard deviations (SD) below the mean body weight (i.e., zero) force [5], with the upward (concentric) phase identified using the instantaneous vertical velocity method as the first positive vertical velocity value identified following the descending (eccentric) phase. The vertical ground reaction force was integrated using the trapezoid method during the eccentric and concentric phases of the jump. The net impulse was calculated independently and summed from the left and right force platforms. Ground reaction forces were directly quantified by integrating the applied force over time (i.e., impulse), which is equivalent to the change in momentum of the body:

J=∫F·dt= Δp

where J = impulse, F = force, dt = time, and Δp = change in momentum.

The take-off velocity was determined by dividing impulse by body mass, and the jump height was calculated using standard equations of motion [41]. To calculate power, the impulse-momentum approach was used. Since the force, mass, and initial velocity conditions were known, the instantaneous velocity could be calculated. The instantaneous power was calculated as force × velocity, and the peak values were determined for the propulsive phase of the CMJ using the following equation:

$$F\left(i\right)\cdot t=m\cdot (v\left(i+1\right)-v(i))$$

$$\Delta v=\frac{F\left(i\right)\cdot t}{m}$$

$$P\left(i\right)=F\left(i\right)\times v(i)$$

where F = force, t = 1/sampling frequency, m = mass, v = velocity, and P = power.

The peak eccentric kinetic energy (KE) developed during the jumps was calculated as follows:

$$KE=\;\frac{1}{2}\cdot \;m\cdot \;v^{2}$$

where m = subject’s mass and v = velocity of the countermovement phase.

### 2.4. Statistical Analyses

Statistical analyses were conducted using SPSS for Windows (v.28; IBM Corp., Armonk, NY, USA). Normality of distribution was examined via Shapiro–Wilk tests, with homogeneity of variance assessed via Mauchly’s tests. Data that failed to meet the assumption of normal distribution were transformed (natural log and square root); where data continued to fail the parametric assumptions (peak power, concentric knee angular velocity), non-parametric tests (Friedman and Wilcoxon tests) were performed. Data that satisfied the parametric assumptions were analyzed using a two-way repeated measures ANOVA (time × 5 [Pre-intervention, 30 s, 4 min, 8 min, 12 min], condition × 2 [v_fast_, v_slow_]) to examine within-subject effects of time and condition, with correction factors (Greenhouse-Geisser) used where sphericity was violated. Where a significant interaction effect was detected, simple main effects analyses (pairwise comparisons) were conducted using Tukey’s LSD to compare each time point to pre-intervention; where no interaction effect was detected, main effects (i.e., data collapsed by time or condition) were examined. Standardized effect sizes (with 95% confidence intervals [CI]) were calculated to examine the magnitude of change. For parametric analyses (ANOVA and paired *t*-tests), partial eta squared (ηp^2^) and Cohen’s *d* (*d*) were calculated, respectively, while for non-parametric analyses (Friedman and Wilcoxon tests), Kendall’s *W* (*W*) and *r* (*r*; the standardized effect size for Wilcoxon’s tests)) were calculated, respectively [42]. Group data that satisfied the parametric assumptions are reported as mean ± SD, whereas data that failed parametric assumptions are reported as median (Md) and interquartile range (IQR); all change data are reported as mean ± SD. Statistical significance for all tests was accepted at *p* < 0.05.

#### Reliability

Between-session reliability was assessed by calculating intraclass correlation coefficients (ICC) using the guidelines of Koo and Li [43], alongside coefficients of variation (CV). Two-way mixed absolute agreement ICCs (ICC_3,1_) revealed good-to-excellent reliability for jump height (ICC = 0.88, CV = 7.5%), kinetic energy (ICC = 0.91, CV = 7.5%), peak power (ICC = 0.92, CV = 6.6%), knee angle (ICC = 0.82, CV = 5.0%), and concentric knee angular velocity (ICC = 0.87, CV = 1.8%), but poor reliability for eccentric knee angular velocity (ICC = 0.46, CV = 9.5%).

## 3. Results

### 3.1. Maximum Countermovement Jump Height

**Figure 1:**
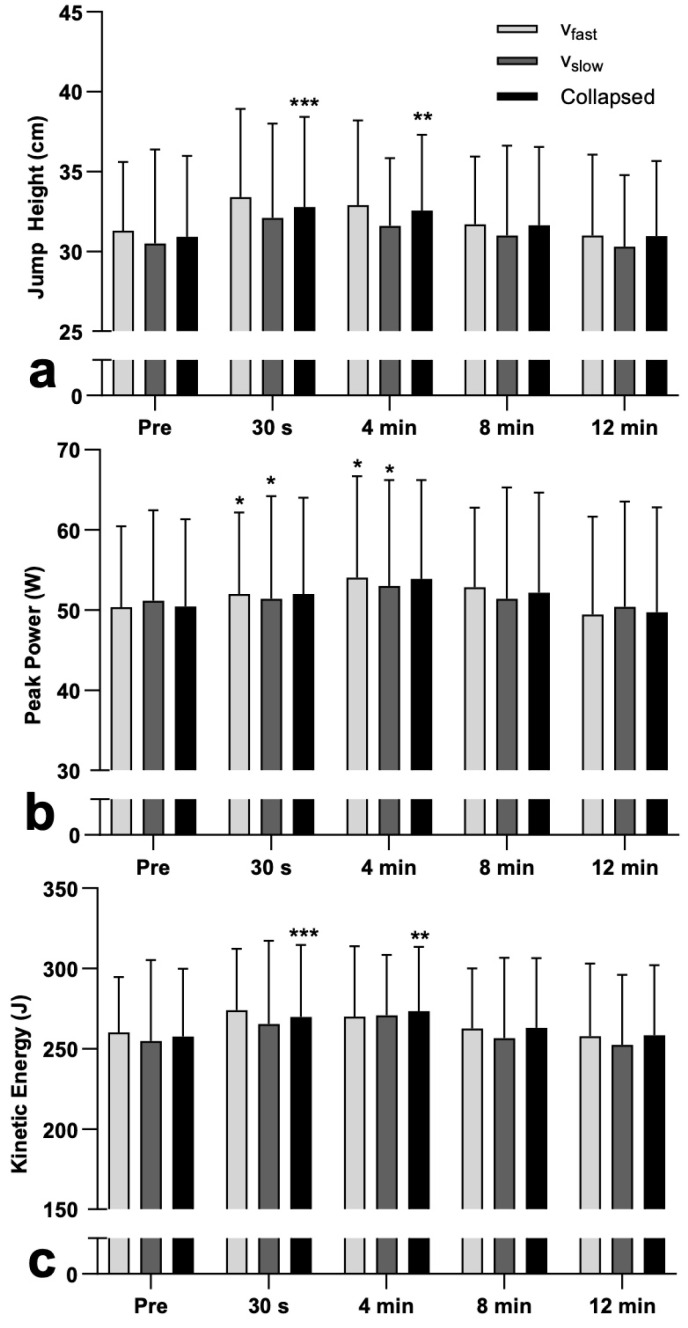
Slow velocity (v_slow_), fast velocity (v_fast_), and collapsed (grouped) outcomes for (**a**) mean ± SD maximum countermovement jump height, (**b**) median ± IQR peak power, and (**c**) mean ± SD kinetic energy presented as a percentage (%) change at each time point during jump trials. Significant increases (collapsed data) in jump height (6.2%), power (3.4–6.2%), and energy (4.9–7.5%) were detected at 30 s and 4 min post-intervention. * *p* < 0.05, ** *p* < 0.01, *** *p* < 0.001. Pre = before intervention.

No significant interaction effect was detected for maximum jump height (F_2.04, 26.54_ = 0.213, *p* = 0.814, ηp^2^ = 0.016), with no main effect of condition (F_1, 13_ = 1.875, *p* = 0.194, ηp^2^ = 0.126) but a significant effect of time (F_4, 52_ = 8.389, *p* < 0.001, ηp^2^ = 0.392) detected. Compared to pre-intervention (collapsed group data), jump height was significantly (see Figure 1a) higher at 30 s (6.2 ± 8.0%, 1.86 ± 2.24 cm) and 4 min (6.2 ± 10.6%, 1.64 ± 2.41 cm) but not at 8 min (2.8 ± 6.4%, 0.73 ± 1.84 cm) or 12 min (0.9 ± 10.3%, 0.05 ± 2.61 cm) (see Table 2).

### 3.2. Peak Power

Wilcoxon tests revealed no significant between-condition differences at any time point (*p* = 0.074–0.433) for peak power. For the v_slow_ condition, Friedman’s test revealed a significant effect of time (χ^2^[4] = 12.514, *p* = 0.014, W = 0.89), with post hoc Wilcoxon tests revealing significantly (see Figure 1b) greater peak power at 30 s (4.0 ± 5.0%, 1.76 ± 1.94 W) and 4 min (6.2 ± 10.9%, 2.33 ± 3.29 W) but not at 8 min (3.4 ± 6.7%, 1.40 ± 3.02 W) or 12 min (4.0 ± 9.7%, 1.40 ± 3.42 W). Similarly, for the v_fast_ condition, Friedman’s test also revealed a significant main effect of time (χ^2^[4] = 11.086, *p* = 0.026, W = 0.79), with peak power significantly greater at 30 s (3.4 ± 6.3%, 1.63 ± 2.67 W) and 4 min (4.1 ± 5.4%, 2.05 ± 2.45 W) but not at 8 min (2.6 ± 5.3%, 1.18 ± 2.47 W) or 12 min (−1.0 ± 5.4%, −0.37 ± 2.60 W).

### 3.3. Kinetic Energy

No significant interaction effect was detected for kinetic energy (F_1.75, 22.80_ = 0.346, *p* = 0.684, ηp^2^ = 0.026), and no main effect of condition (F_1, 13_ = 1.475, *p* = 0.246, ηp^2^ = 0.102). However, a significant effect of time (F_4, 52_ = 7.467, *p* < 0.001, ηp^2^ = 0.365) was detected. Compared to pre-intervention (collapsed group data), kinetic energy was significantly (see Figure 1c) greater at 30 s (4.9 ± 6.2%, 12.27 ± 14.25 J) and 4 min (7.5 ± 16.2%, 15.87 ± 25.68 J) but not at 8 min (2.3 ± 5.4%, 5.49 ± 13.19 J) or 12 min (0.8 ± 9.7%, 0.76 ± 19.90 J).

### 3.4. Knee Angle

**Figure 2:**
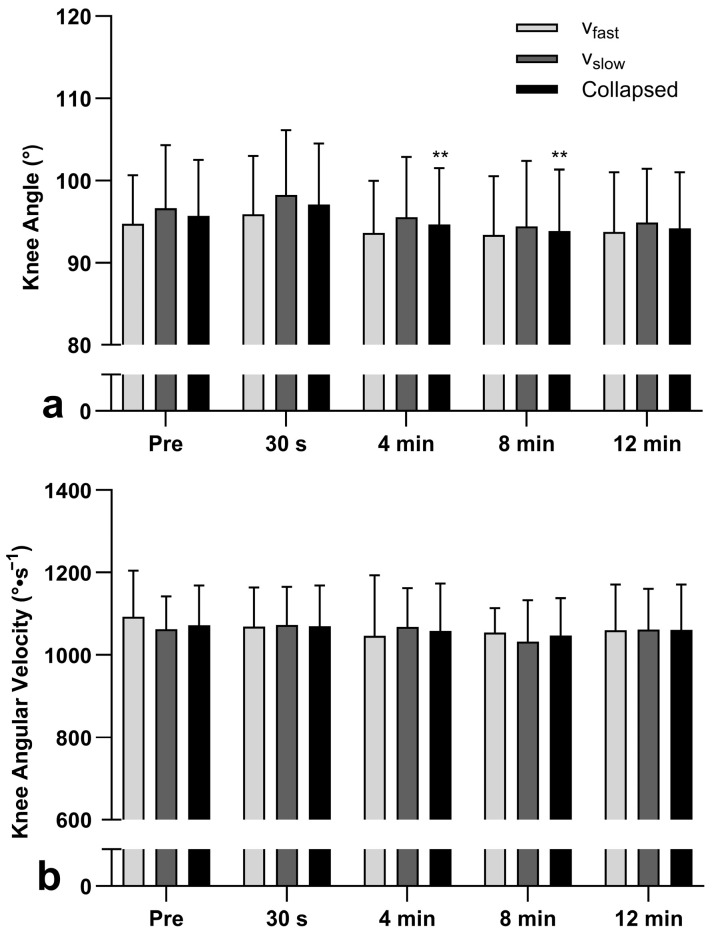
Slow velocity (v_slow_), fast velocity (v_fast_), and collapsed (grouped) outcomes for (**a**) mean ± SD maximum countermovement knee flexion angle and (**b**) median ± IQR concentric knee angular velocity at each time point during jump trials. No significant change (collapsed data) in knee angle or angular velocity was detected at any time point compared to pre-intervention. ** *p* < 0.01, significantly different from 30 s post-intervention (Pre).

No significant interaction effect was detected for maximum knee angle (F_4, 52_ = 0.085, *p* = 0.987, ηp^2^ = 0.007), and no main effect of condition (F_1, 13_ = 2.378, *p* = 0.147, ηp^2^ = 0.155). However, a significant effect of time (F_4, 52_ = 3.407, *p* = 0.015, ηp^2^ = 0.208) was detected. Compared to pre-intervention (collapsed group data), maximum knee angle was not significantly (see Figure 2a) different at any time point (30 s: 1.6 ± 5.6%, 1.6 ± 5.3°, 4 min: −0.9 ± 5.2%, −0.6 ± 5.2°, 8 min: −1.8 ± 5.3%, −1.8 ± 5.1°, 12 min: 1.4 ± 6.1%, −1.5 ± 5.9°).

### 3.5. Knee Angular Velocity

Wilcoxon tests revealed no significant between-condition differences at any time point in concentric knee angular velocity (*p* = 0.249–0.638). Friedman’s test revealed no main effects of time (see Figure 2b) for v_slow_ (χ^2^[4] = 8.457, *p* = 0.076, W = 0.60) or v_fast_ (χ^2^[4] = 5.543, *p* = 0.236, W = 0.40) in concentric knee angular velocity. Eccentric knee angular velocity was not statistically analyzed due to poor within-session reliability (ICC = 0.46)

## 4. Discussion

The primary aim of the present study was to determine and compare the magnitude and time course of changes in CMJ performance after slow (v_slow_) and fast (v_fast_) eccentric-phase (downward) velocity squat conditioning activities following a comprehensive, task-specific warm-up. Both v_slow_ and v_fast_, using a moderate load of 70% 1-RM and with the concentric phase performed with maximal effort, demonstrated significant increases in jump height at 30 s (6.2 ± 8.0%) and 4 min (6.2 ± 10.6%) but not at 8 min or 12 min post-intervention, partly confirming the first hypothesis. These findings are consistent with previous research showing that moderate (70%) and heavy (93%) 1-RM back squat exercises can significantly increase countermovement jump performance at 4 min with a return to baseline at 8 min and 12 min [44]. Whereas others [5] have reported a lack of jump performance enhancement following a heavy free-weight back squat conditioning activity at 85% 1-RM performed after a comprehensive, task-specific warm-up, as performed in the present study. Mina et al. [5] suggested that high-intensity contractions might only enhance performance when following a limited and insufficient warm-up. Such a lack of PAPE after a comprehensive warm-up was also identified in a recent meta-analysis [9], which consistently showed that extensive or high-volume warm-ups may reduce or remove PAPE. As comprehensive warm-ups can induce acute neuromuscular and physiological adaptations to enhance performance (PAPE), incorporating a conditioning activity within a comprehensive warm-up protocol may provide negligible additional benefits and could potentially impair performance during the initial minutes following a conditioning activity completion. In fact, even without comprehensive warm-up, PAPE might not always be observed [45]. The inconsistencies in PAPE responses in previous research [5,14,25,45] may partly be explained by variable interactions of fatigue, motor pattern perseveration (interference), potentiation, and their cumulative influence on subsequent performance [3]. It may also reflect variability in PAPE responses, both between individuals and within individuals across time points [9].

Although a PAPE was observed in the intervention conditions, v_slow_ and v_fast_ both enhanced CMJ performance and eccentric force capabilities of the active musculature equally at 30 s and 4 min, evidenced by increases in jump height, peak power and kinetic energy, with no significant differences detected between conditions. Therefore, the second hypothesis that the slow (v_slow_) eccentric velocity squat CA may result in greater improvements in subsequent CMJ height than the fast (v_fast_) CA is rejected. Both conditions, not only could be useful for enhancing performance, but the result also shows that eccentric-phase velocity, and thus, the kinetic energy available for storage in series elastic elements at the end of the eccentric (downward) phase, may not influence concentric PAPE magnitude, at least when using moderate-load (70%) back squats to enhance CMJ performance. Thus, the first hypothesis can be partially accepted. These effects may indicate that the potential acute positive and negative adaptations to the conditioning activities had similar overall effects on both CMJ performance and the movement patterns used to perform the CMJ. Therefore, possibly v_fast_ might have enhanced neural drive and motor unit recruitment due to high-velocity eccentric loading and rapid force production [31,32], while also minimizing fatigue. This may have contributed to the enhancement in subsequent performance by acutely increasing musculotendinous stiffness and improving the efficiency of the stretch-shortening cycle function, thereby facilitating greater force transmission and rate of force development during the CMJ [31]; however, it may have also provided less time for motor learning or imposed greater technical demands. In contrast, v_slow_ may have increased time under tension, meaning the muscles remained actively loaded for longer during each repetition. This extended contraction can raise muscle temperature and metabolic stress, which may enhance the stimulus for muscular adaptation [17,18]. The slower eccentric phase may have allowed more opportunity for sensory feedback allowing the nervous system to refine movement patterns, potentially improving motor learning and technique control; but also may have led to greater fatigue and transient reductions in power output [17]. The data collected in the present study do not allow testing of these hypotheses. Nonetheless, it might be of interest in future research to determine whether a conditioning activity that integrates both fast and slow eccentric phases elicits additive or synergistic adaptations beyond those observed when each modality is applied in isolation.

While the test-specific practice should have optimized jump performance, it is possible that the lower volume of contractions and substantial between-jump rest allowed temperature to decrease slightly. Temperature is a critical factor influencing PAPE, since the increased rate of force development and shortening velocity is enhanced in both fast and slow twitch fibers by temperature increases [12,46,47]. The rise in muscle temperature may have been greater in the v_slow_ condition, in which muscles underwent a relatively prolonged eccentric contraction [25,26,27]. In contrast, the v_fast_ condition likely involved later muscle activation and a higher rate of force development (RFD), minimizing the extent of muscle lengthening once activated [31,32]. However, the prolonged time under tension associated with slow eccentric contractions can possibly induce fatigue and mechanical stress [17]. In the present study, the intended contraction velocity did not influence performance, as no significant differences were identified between the v_slow_ and v_fast_ conditions. The novelty of this study is that while the net performance outcome remains stable, the underlying driver of PAPE may shift from thermogenic (v_slow_) to neural (v_fast_) depending on the contraction duration. This suggests that the “intent” to move at a specific velocity may be less critical than the total work (i.e., load) performed. It was not possible to measure muscle temperature in the present study, and this should be a goal of future research examining the variability of PAPE with interventions.

An interesting observation in the present study was that both PAPE and movement pattern alterations were observed at the earliest time point (30 s) and at 4 min post-CA, but were not detectable by 8 min post-CA. Although within-subject factors (e.g., training status, muscle fiber composition) and study design elements (e.g., type of conditioning activity, performance task) can influence the PAPE time course [3,48], it has been reported to peak at 7 to 10 min [12] after a moderate-intensity dynamic conditioning (60–84% 1-RM) and 30 s to 12 min [5,49] after high-load conditioning (85–100% 1-RM). Of note, however, is that Golas et al. [48] found that the optimum recovery times among well-trained athletes (i.e., basketball, luge and athletic throws) for activities such as jumping, throwing and pushing varied according to the athlete’s muscular strength, training status, and muscle fiber type distribution, confirming that PAPE recovery periods cannot be generalized across individuals. This rapid onset of PAPE in the present study is consistent with findings of Mina et al. [5], who reported CMJ enhancement at all time points from 30 s to 12 min after weighted squat jumps performed with elastic band resistance after a comprehensive warm-up. Such findings have important practical implications, as previous research demonstrating delayed responses has typically indicated optimal time windows averaging 4–10 min post-conditioning [12,50].

Eccentric velocities during the CA were prescribed as v_slow_ and v_fast_ but were not directly recorded, as the primary focus of this study was on subsequent performance outcomes rather than on the mechanical characteristics of the conditioning stimulus. Therefore, this is a limitation of the present study, and future research is required to directly quantify eccentric velocity during the conditioning activity. Further, it will be of interest in future research to test the effects of including lifts performed with both slower- and faster-velocity eccentric and concentric phases as a conditioning activity. To comprehensively describe the impacts of squat velocity, in both the concentric and eccentric phases of conditioning activities, further research is needed to explore whether similar effects are observed across a range of velocities and exercises (e.g., bench press, deadlift). Additionally, although the study was adequately powered, the relatively small sample size (*n* = 14) may have limited the ability to detect small differences between conditions. Another limitation of the present study is that only male participants were included, which may limit the generalizability of the findings across sexes. Therefore, investigating the response to these protocols across different training status populations, such as elite athletes and novice individuals, and across specific age groups may provide a more comprehensive understanding of the practical implications and applications of these findings.

### Practical Application

Velocity of the downward (eccentric) phase of a squat lift conditioning activity does not appear to influence the magnitude or temporal profile of CMJ performance enhancement, at least when performed after a comprehensive, task-specific warm-up [3,45]. The possibility exists therefore, that the potential benefits and drawbacks of faster versus slower eccentric velocity equalize between the conditions; hence, neither condition emerges as superior [24]. Coaches and athletes can use either fast or slow eccentric squat velocities as part of a conditioning activity using a 30 s to 4 min window to maximize benefits, allowing coaches to select based on athlete preference, technical competency, or specific training goals. However, both slow and fast eccentric-velocity squat conditioning activities performed with a moderate load (70% 1-RM) can acutely enhance countermovement jump performance, although these effects appear short-lived (up to ~4 min). For practitioners, this suggests that incorporating either slow- or fast-eccentric squat variations into pre-training or pre-competition warm-up routines may provide a brief window of enhanced explosive performance, but these benefits are unlikely to persist beyond 8 min [12]. These results also highlight that extensive warm-ups themselves provide substantial performance benefits, which may limit or mask additional potentiation effects. Therefore, strength and conditioning professionals should consider both the duration and structure of warm-up and conditioning activities when aiming to optimize readiness for explosive tasks, such as jumping, sprinting, or change of direction. Future applications may involve tailoring conditioning activity intensity, velocity, and recovery interval to the individual athlete’s training status, strength levels, and competition demands.

## 5. Conclusions

To conclude, the inclusion of v_slow_ and v_fast_ eccentric-velocity squat conditioning activities performed with a moderate load (70% 1-RM) can acutely enhance countermovement jump performance above warm-up alone, although these effects appear short-lived (up to ~4 min). The increase in jump performance up to 4 min, as previously observed, may be an effect of the moderate free-weight load alone without the influence of the intended eccentric-phase velocity [45]. The present findings suggest that conditioning activities may elicit more immediate, although potentially shorter-lasting, performance benefits when preceded by a comprehensive task-specific warm-up. Importantly, it should also be noted that the warm-up itself would likely have provided a substantial performance benefit, and the results show that performance returns to this prior, already enhanced, performance level. It is likely that it would take much longer to return to pre-warm-up levels, as observed in other studies where no warm-up, or a relatively short, low-intensity warm-up, is imposed before baseline testing [9]. Such a hypothesis should be explicitly tested in future studies. This timing consideration is particularly relevant for practitioners developing pre-training and pre-competition routines where the balance between the positive effects of potentiation and deleterious effects of fatigue and motor pattern interference, and thus subsequent performance enhancement, must be carefully managed.

## Figures and Tables

**Table 1:** Study design timeline.

Task	Time (min)
7 min light jogging (7.5 km·h^−1^)	0.0–5.0
5 unloaded squats (2 s/2 s)	5.0–6.0
5 unloaded squats (1 s/1 s)	6.0–7.0
5 CMJs (70%)	7.5–8.5
Single CMJ every 30 s (100%)	9.0–11.0
CMJ Test 1	13.0–13.5
Back squats (v_fast_ or v_slow_)	14.5–15.0
CMJ Tests (2–4)	15.5, 19.5, 23.5, 27.5

CMJ, countermovement jump; v_fast_, fast velocity during eccentric phase; v_slow_, slow velocity during eccentric phase.

**Table 2:** Effect size and 95% confidence intervals at 30 s, 4 min, 8 min, and 12 min post-intervention compared to pre-intervention for all outcome variables.

Variable	Group	30 s ES (CI)	4 min ES (CI)	8 min ES (CI)	12 min ES (CI)
Countermovement jump height	v_slow_	*d =* 0.87 (0.24, 1.47)	*d =* 0.55 (−0.03, 1.10)	*d =* 0.39 (−0.16, 0.93)	*d =* 0.05 (−0.48, 0.57)
	v_fast_	*d =* 0.82 (0.20, 1.41)	*d =* 0.82 (0.20, 1.42)	*d =* 0.39 (−0.16, 0.92)	*d =* −0.01 (−0.53, 0.51)
	Collapsed	*d =* * 0.83 (0.40, 1.26)	*d =* * 0.68 (0.26, 1.09)	*d =* 0.40 (0.01, 0.78)	*d =* 0.02 (−0.34, 0.38)
Peak power	v_slow_	* r = 0.32 (0.05, 0.60)	* r = 0.32 (0.05, 0.60)	*r* = 0.04 (−0.25, 0.33)	*r* = 0.11 (−0.17, 0.40)
	v_fast_	* r = 0.32 (0.05, 0.60)	* r = 0.38 (0.12, 0.65)	*r* = 0.18 (−0.10, 0.47)	*r* = 0.03 (−0.26, 0.32)
	Collapsed	NA	NA	NA	NA
Kinetic energy	v_slow_	*d =* 0.83 (0.20, 1.43)	*d =* 0.56 (−0.13, 1.12)	*d =* 0.37 (−0.18, 0.90)	*d =* 0.05 (−0.47, 0.58)
	v_fast_	*d =* 0.88 (0.24, 1.48)	*d =* 0.88 (0.24, 1.48)	*d =* 0.46 (−0.09, 1.01)	*d =* 0.02 (−0.50, 0.55)
	Collapsed	*d =* * 0.86 (0.42, 1.29)	*d =* * 0.62 (0.21, 1.02)	*d =* 0.42 (0.03, 0.80)	*d =* 0.04 (−0.33, 0.41)
Knee angle	v_slow_	*d =* 0.28 (−0.26, 0.81)	*d =* 0.21 (−0.73, 0.33)	*d =* −0.32 (−0.85, 0.22)	*d =* −0.31 (−0.84, 0.24)
	v_fast_	*d =* 0.23 (−0.31, 0.76)	*d =* −0.18 (−0.71, 0.35)	*d =* 0.46 (−1.01, 0.10)	*d =* −0.21 (−0.73, 0.33)
	Collapsed	*d =* 0.26 (−1.12, 0.64)	*d =* −0.20 (−0.57, 0.18)	*d =* −0.36 (−0.74, 0.02)	*d =* 0.26 (−0.61, 0.12)
Knee angular velocity	v_slow_	*r* = 0.53 (0.28, 0.77)	*r* = 0.13 (−0.16, 0.41)	*r* = 0.32 (0.05, 0.59)	*r* = 0.36 (0.09, 0.63)
	v_fast_	*r* = −1.37 (0.05, 0.59)	*r* = 0.43 (0.17, 0.69)	*r* = 0.53 (0.28, 0.77)	*r* = 0.23 (−0.05, 0.51)
	Collapsed	NA	NA	NA	NA

Acronyms: ES = effect size, CI = confidence interval, v_slow_ = intended slow-velocity eccentric phase, v_fast_ = intended fast-velocity eccentric phase, *d* = Cohen’s D, *r* = non-parametric effect size, * significantly different from pre-intervention, NA = not applicable as collapsed group data were not analyzed (data did not satisfy parametric assumptions).

## Data Availability

The data of the study are available from the corresponding author upon reasonable request, subject to ethical considerations.

## Abbreviations

The following abbreviations are used in this manuscript:

1-RM	One repetition maximum
CMJ	Countermovement Jump
CA	Conditioning Activity
CI	Confidence interval
ES	Effect size
KE	Kinetic energy
N	Ground reaction force
PAP	Post-activation potentiation
PAPE	Post-activation performance enhancement
RFD	Rate of force development
SD	Standard deviation
SSC	Stretch shortening cycle
v_slow_	Slow velocity
v_fast_	Fast velocity
